# Lessons Learned from a Single Institution’s Eight Years of Experience with Early Cleft Lip Repair

**DOI:** 10.3390/medicina59101741

**Published:** 2023-09-28

**Authors:** Idean Roohani, Collean Trotter, Pasha Shakoori, Tayla A. Moshal, Sasha Lasky, Artur Manasyan, Erin M. Wolfe, William P. Magee, Jeffrey A. Hammoudeh

**Affiliations:** 1 Division of Plastic and Maxillofacial Surgery, Children’s Hospital Los Angeles, Los Angeles, CA 90027, USA; roohani@usc.edu (I.R.); moshal@usc.edu (T.A.M.); sashalasky4@gmail.com (S.L.); wmagee@chla.usc.edu (W.P.M.III); 2 Keck School of Medicine, University of Southern California, Los Angeles, CA 90089, USA; amanasya@usc.edu; 3 Division of Plastic and Reconstructive Surgery, Keck School of Medicine of USC, Los Angeles, CA 90033, USA; pasha.shakoori@med.usc.edu (P.S.); erin.wolfe@med.usc.edu (E.M.W.)

**Keywords:** cleft lip, cleft palate, nasoalveolar molding, neonate, cleft lip and palate, children, infants, humans, maxilla

## Abstract

*Background and Objectives*: The traditional approach in managing wide cleft lip deformities involves presurgical nasoalveolar molding (NAM) therapy followed by surgical cleft lip repair between three and six months of age. This institution has implemented an early cleft lip repair (ECLR) protocol where infants undergo primary cleft lip repair between two and five weeks of age without NAM. This study aims to present this institution’s ECLR repair protocol over the past eight years from 188 consecutive patients with unilateral or bilateral CL/P deformity. *Materials and Methods*: Retrospective review was conducted at Children’s Hospital Los Angeles evaluating patients who underwent ECLR before three months of age and were classified as American Society of Anesthesiologists (ASA) class I or II from 2015–2022. Anthropometric analysis was performed, and pre- and postoperative photographs were evaluated to assess nasal and lip symmetry. *Results*: The average age at cleft lip repair after correcting for gestational age was 1.0 ± 0.5 months. Mean operative and anesthetic times were 120.3 ± 33.0 min and 189.4 ± 35.4, respectively. Only 2.1% (4/188) of patients had postoperative complications. Lip revision rates were 11.4% (20/175) and 15.4% (2/13) for unilateral and bilateral repairs, respectively, most of which were minor in severity (16/22, 72.7%). Postoperative anthropometric measurements demonstrated significant improvements in nasal and lip symmetry (*p* < 0.001). *Conclusions*: This analysis demonstrates the safety and efficacy of ECLR in correcting all unilateral cleft lip and nasal deformities of patients who were ASA classes I or II. At this institution, ECLR has minimized the need for NAM, which is now reserved for patients with bilateral cleft lip, late presentation, or comorbidities that preclude them from early repair. ECLR serves as a valuable option for patients with a wide range of cleft severity while reducing the burden of care.

## 1. Introduction

Recent advancements in neonatology have facilitated surgical correction of various congenital anomalies where each year, more than 1.5 million neonates undergo general anesthesia for a surgical procedure [1]. Earlier surgical correction of cleft lip is one such procedure where higher plasticity of the maxilla and nasal cartilaginous structures in the neonatal period can result in improved reconstructive outcomes, in addition to improved feeding and weight gain earlier in life [2,3,4]. Despite these advancements, surgical correction of cleft lip, the seventh most common congenital anomaly globally, is not routinely performed in the neonatal period. This is primarily due to the technically challenging nature of the procedure during this period, coupled with limited healthcare infrastructure providing neonatal anesthesia and restricted accessibility to care.

In the cleft care paradigm, cleft lip nasal deformities are commonly repaired after three months of age. Further dissecting the rationale behind this timeline, this consensus was based on the “Rule of 10 s”, as described by Wilhelmsen and Musgrave in 1966 and later modified by Millard in 1976 [5,6,7,8]. This precedent recommends that for patients to undergo safe cleft lip repair, the patient would have to weigh greater than 10 pounds, have a hemoglobin level greater than 10 g/dL, and have a white blood cell count greater than 10,000 cells/μL [6]. However, significant advances over the past five decades have improved preventive measures, access to prenatal and neonatal diagnosis and treatment, nutritional optimization, and anesthetic and antiseptic techniques [9,10,11,12]. Ultimately, these advances have resulted in improved outcomes, resulting in reduced infant mortality rates in the United States from 23.45 deaths in every 1000 live births in 1966 to 5.48 deaths in every 1000 live births in 2023 [13]. These advancements in healthcare services have resulted in an increase in life expectancy in the U.S. from 70.26 years in 1966 to 79.11 years in 2023 [13]. Considering these improved trends in healthcare access, technologies, and outcomes over the past few decades, many clinicians have attempted to improve cleft repair outcomes by learning from past lessons.

Historically, some craniofacial surgeons have advocated for cleft lip repair in healthy newborns as early as a few weeks of life [9,10,14,15]. In 1967, Canon et al. suggested a financial, social, and nutritional advantage to cleft lip repair completed 24 h after birth in healthy neonates, with two to four weeks of life being acceptable if an immediate surgical repair was not feasible [16]. In 1983, Bromley et al. documented the repair of unilateral and bilateral cleft lips as early as one week of age with an overall 11.1% morbidity and 0% mortality rate over 15 years [17]. Weatherly-White et al. reported similar lip revision rates between patients undergoing early and standard cleft lip repair [11]. Compared to those who underwent cleft lip repair at a later stage, patients undergoing earlier repair have demonstrated improved feeding and comparable complication rates within traditional best practices [4,10,11,18]. While not commonly performed, it is important to acknowledge that some surgeons may close the lip and palate simultaneously at an age relatively earlier than traditional palatoplasty, some as early as 6 weeks of life [19].

Moving from traditional methods, contemporary techniques have improved aesthetic and functional results of primary surgical procedures. Early cleft lip nasal deformity repair takes advantage of inherent neonatal plasticity, which results from the persistent circulation of maternal estrogen in the neonatal tissue that is still present soon after birth. These high estrogen levels during the neonatal period facilitate the pliability of the facial hard and soft tissues, providing an ideal molding environment to decrease the alveolar cleft width. This subsequently reduces tension during cleft lip repair, which is particularly advantageous for patients with cleft lip and palate. Moreover, this neonatal milieu allows molding the malleable nasal cartilage within the first six weeks of life, as originally analogously employed by ear molding therapy in 1989 [20]. Estrogen stimulates the production of hyaluronic acid and transforming growth factor-beta (TGF-β) [21,22,23], which promote better wound healing, less perceptible scarring, and decreased need for lip revision surgery.

Parallel to the evolution of various cleft lip repair techniques, surgeons appreciated the importance of nasal correction and the interplay of the bony foundation of the maxilla, cartilaginous structures of the nose, and the interplay of these components in successful cleft lip nasal deformity repair. As demonstrated by McComb et al., primary surgical repair of the cleft lip nasal deformity can be performed at the time of cleft lip repair, which allows for early restoration of the nasal shape and can obviate the need for later cleft lip nasal reconstruction [24]. Various studies have demonstrated that early manipulation of the nasal cartilage does not interfere with growth [25]. It serves to reason that early surgical correction of the cleft lip nasal deformity may also improve conformation by arresting the progressive nasal deformity, which has been noted to occur in the neonatal period [26]. Interestingly, the surgical correction of clefts with a Simonart’s band requires less nasal and lip revisions, in part by directing the non-cleft and cleft segments closer to each other prior to surgical repair [27]. Surgical adjuncts of neonatal repair, such as the simultaneous insertion of nasal retainers to address nasal deformities, have been described to correct nostril deformity and further improve nasal symmetry [28]. These adjuncts are more effective in the neonatal period, where maternal circulating levels of estrogen are higher compared to later periods of traditional repair, an integral concept.

Additionally, the bony foundations of the maxilla and its corresponding deformity are important considerations while planning a cleft lip nasal deformity repair. Nasoalveolar molding (NAM), also referred to as presurgical NAM (PNAM), takes advantage of this concept and has traditionally been utilized in patients with wide complete unilateral CL/P (UCL/P) and bilateral CL/P (BCL/P) to optimize maxillary segment alignment as well as improving soft tissue configuration to facilitate repair [29]. This presurgical orthodontic device takes advantage of the aforementioned plasticity. Attempts at total nonsurgical correction of the cleft nasal deformity through presurgical nasal molding have been described with some success [26,28].

The advantages of NAM are well-known; however, NAM therapy can significantly burden on the healthcare system, and the patient’s family and support system [29,30,31]. Caregivers must travel on average 65 miles for appliance adjustment appointments as often as weekly [29,32]. Depending on the cleft severity, 13 to 22 office visits have been reported for NAM care [29]. Proper appliance positioning and adherence to surgical taping protocols can be challenging. In contrast, ECLR offers a safe, effective, and lower-cost alternative for cleft lip repair which eliminates the need for NAM, thereby reducing the burden of care. Despite this institution’s history as one of the largest NAM programs nationwide, currently, ECLR is being offered at a 98% acceptance rate, creating a paradigm shift where primary cleft lip repair is being performed in the first two to five weeks of life [3,4]. Thus, this cohort of patients undergoing earlier cleft lip repair is the most extensive in comparison to other institutions and may help to elucidate the social, financial, aesthetic, and functional impact that ECLR will have on cleft care. This institution has preliminarily demonstrated the safety and efficacy of the described ECLR protocol [3].

This study evaluated a series of 188 consecutive patients with CL/P who underwent ECLR at a pediatric tertiary hospital. The purpose was to present this institution’s experience of ECLR over the past eight years by assessing the postoperative complications, secondary revisions, and anthropometrics of this patient cohort.

## 2. Materials and Methods

### 2.1. Study Participants

A retrospective review, approved by the institutional review board (IRB#: CCI-14-00001), was performed evaluating patients who underwent ECLR before 3 months of age from 2015 to 2022 at Children’s Hospital Los Angeles was performed. All patients received care at an American Cleft Palate-Craniofacial Association-certified cleft care center by the Division of Plastic and Maxillofacial Surgery. A total of 188 patients met inclusion criteria. Exclusion criteria included patients with an American Society of Anesthesiologists (ASA) class III or higher or those diagnosed with a craniofacial syndrome. These groups of patients were excluded undergoing earlier repair as it is recommended that patients with concerning comorbidities are operated on with a traditional timeline. The families were then offered traditional cleft lip repair once these patients’ condition was deemed medically stable.

### 2.2. Data Collection

Electronic medical records were reviewed to extract patient demographics (i.e., age, sex, race, Hispanic ethnicity), ASA classification, gestational age, weight at surgery, and cleft characteristics (i.e., cleft phenotype, laterality, severity). Racial groups included White/Caucasian, Asian, Black/African American, American Indian or Alaska Native, and Native Hawaiian or Other Pacific Islander; although, no families in this cohort reported on the latter two and thus were not included in the results. Cleft severity was determined based on clinical examination and noted in the medical records as the patient’s preoperative diagnosis; this included three classifications: (1) microform (i.e., incomplete lip separation with distortion but no separation of the vermillion border), (2) incomplete (i.e., lip separation through the vermillion border but an intact nasal sill), or (3) complete (i.e., complete separation of lip and nasal sill) [33]. Perioperative data included anesthesia time, operative time, and length of hospital stay (LOS). Postoperative complications were categorized as unplanned readmission, complications associated with nasal stents, wound infections, failure to thrive, perioperative mortality, and major anesthetic complications (i.e., code events, intraoperative respiratory failure, and aborted operations). The necessity, timing, and indication for cleft lip revision were also abstracted. Cleft lip revisions were then categorized as major or minor. A major lip revision was defined as a complete takedown and recreation of the original defect and repair of the cleft lip deformity. A minor lip revision was defined as indicated surgical intervention to address abnormal scarring, vermillion/white roll asymmetry, wet–dry junction asymmetry, and excess intraoral tissue.

### 2.3. Anesthesia Protocol

**Figure 1:**
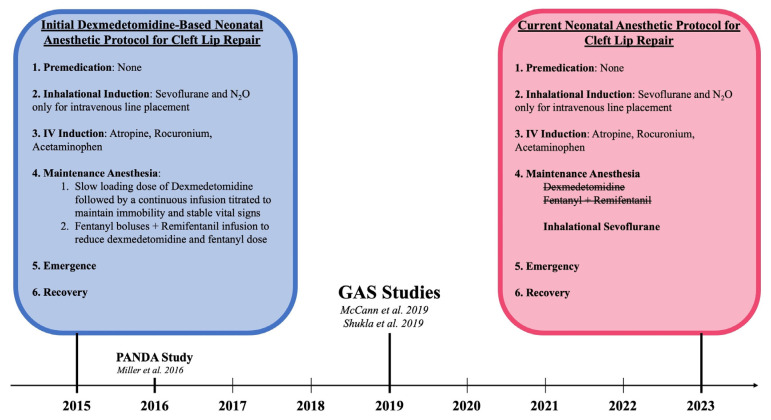
Paradigm Shift in the Anesthesia Protocol for Early Cleft Lip Repair in relation to pivotal anesthesia studies: McCann et al. [34], Shukla et al. 2016 [35], and Miller et al. [36].

All patients underwent cleft lip repair under general anesthesia while monitored by two board-certified physician members of the Department of Anesthesia at a Level I pediatric tertiary hospital. Initially, this protocol included sevoflurane for inhalation induction followed by a continuous infusion of dexmedetomidine and remifentanil for maintenance anesthesia. With consideration of the subsequently performed GAS (General Anesthesia compared to Spinal Anesthesia) and PANDA (Pediatric Anesthesia and Neurodevelopmental Assessment) trials that demonstrated the safety of general endotracheal anesthesia (GETA) use in neonates, this institution transitioned to the standard anesthesia protocol for cleft lip repair [34,35,36]. After careful consideration and discussion with the pediatric anesthesia department, titrated intravenous dexmedetomidine and remifentanil were replaced by inhalation anesthetic utilizing sevoflurane for maintenance anesthesia. This shift in the anesthesia protocol is illustrated in Figure 1.

### 2.4. Operative Technique

All cases of unilateral cleft lip were repaired using a modified subunit technique.

Presurgical Markings

**Figure 2:**
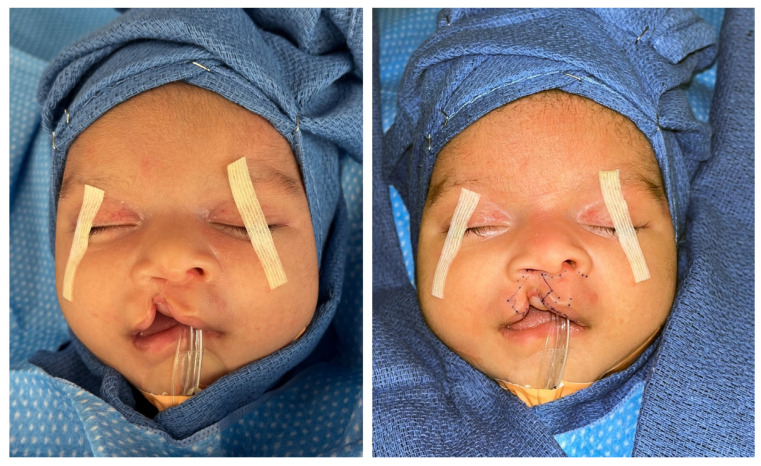
Preoperative photographs without surgical markings (**left**) and with surgical markings (**right**).

1. The anatomical landmarks of the lip and nose were delineated.
  a. Lip markings included:
    i. - Height and depth of Cupid’s bow, lip length, wet–dry vermillion junction and the white roll.
  b. Nasal markings included:
    i. - Columellar base, height of the philtral columns, nasal sill, alar bases, and the skin overlying the medial and lateral footplates of the lower lateral cartilage.
  c. Preoperative surgical markings are illustrated in Figure 2.

Cleft Lip Repair

2. The total lip length was measured to allow us to design the flap.
  a. On the medial element, the lip length and the deficiency were measured.
  b. The lateral lip elements were delineated where the cutaneous aspect of the white roll began to converge with the vermillion-mucosal junction (i.e., Noordhoff point). A triangular-based flap was designed to allow for inset into the medial component.
3. Local anesthetic was administered via infraorbital nerve block in marked structures to avoid the distortion of native anatomy.
4. The initial incision was made using a 67 Beaver blade (Beaver-Visitec International, Abington, Oxfordshire, UK), along with a no. 15-blade for the mucosal side. The medial element was incised through the white roll and resected.
5. The orbicularis muscle was then identified and separated from the overlying dermis using a no. 69 Beaver blade. The incision was then carried cephalad to the base of the columella to release the aberrant attachments of the orbicularis muscle. On the lateral element, the laterally based triangle flap was incised meticulously to preserve the orbicularis muscle through the nonviable element of the cleft.
6. The aberrant attachments of the orbicularis muscle on the lower lateral cartilage were released, followed by resection of the cleft remnant off the lateral element.
7. A vestibular incision on the lateral element was created, initially perpendicular to the mucosa followed by supraperiosteal dissection.
8. The dissection continued cephalad to the nasal pyriform, where the residual nasal sill component of the cleft element was identified and ultimately resected.

Tip Rhinoplasty

9. A marginal incision was created on the cleft nasal side extending in a supra-cartilaginous fashion to the nasal radix.
10. After identifying the aberrant lower lateral cartilage, the cartilage was dissected and mobilized, with the removal of interdomal fat to further allow for cartilage elevation.
11. The anterior nasal spine was repositioned to midline and the nasal floor was reconstructed.
12. A modified McComb suture was then placed, extending from the upper lateral cartilage on the non-cleft side to the lower lateral cartilage on the cleft side.
13. A semi-rigid nasal stent was secured and left in place for four to six weeks postoperatively for cleft nasal cartilage molding.

### 2.5. Postoperative Care

Instructions in terms of postoperative surgical site care are provided to the parents, emphasizing the importance of nasal stent patency and meticulous hygiene to ensure residue accumulation in stent lumens is prevented. Parents are recommended to gently clean around the outside of the nasal stents and the surgical site two to three times per day using a clean cotton swab and a 1:1 mixture of hydrogen peroxide and saline while nasal stents are in place. Following removal, families are recommended to avoid direct sun exposure to the surgical site for one year postoperatively. Additionally, scar massage should be performed three times per day for one year following surgical repair with an over-the-counter photoprotective topical product with a sun protection factor of 30 or higher, which has also been noted by other authors to optimize scar healing and aesthetic outcomes [37].

### 2.6. Photogrammetric Analysis and Inclusion Criteria

Photographs were taken at two different time points: (1) before early cleft lip repair (i.e., preoperative) and (2) within 1 year after surgical correction of the cleft lip (i.e., postoperative). Patients with unilateral cleft lip and nasal deformity were included in the anthropometric analysis if they had all four photographs described previously with the patient in the center of the frame, without nasal stents or lip adhesives, and taken in repose without lip or nostril manipulation. Cases of bilateral cleft lip were excluded from this photogrammetric analysis. Frontal views were used to abstract measurements of the lip length and commissure length, basal views were used to abstract measurements of nostril width and nostril breath.

### 2.7. Anthropometric Measurements

Anthropometric analysis was performed to assess the improvement in nasolabial symmetry postoperatively using Mirror Medical Imaging Software Version 7.5.11 (Canfield Scientific, Inc., Parsippany, NJ, USA). The following standardized anthropometric measurements were obtained on both the cleft and non-cleft side for patients included in the analysis:

- *Lip length*—the distance from the lateral aspect of the columellar base to the ipsilateral peak of Cupid’s bow.
- *Commissure length*—the distance from the labial commissure to the trough of Cupid’s bow.
- *Nostril width*—the distance from the columellar base at its midpoint to the inner nostril at its most inferolateral point).
- *Nostril breadth*—the distance from the lateral aspect of the columellar base to the widest point of the nasal alae.

In order to assess nasal and labial symmetry, symmetry ratios were calculated by dividing the absolute measurement of the cleft side by that of the contralateral (non-cleft) side, with a value of 1.0 indicating ideal symmetry. To quantify the degree of variance from ideal symmetry, the observed symmetry ratio was subtracted by the ideal symmetry ratio (1.0) was calculated.

### 2.8. Statistical Analysis

Descriptive statistical analysis was performed for all collected variables. The sample was thoroughly analyzed to identify any instances of missing data across all collected variables, of which none were detected. Scatter plots and descriptive statistics were used to assess for outliers, revealing no noteworthy observations. Categorical variables were expressed as frequencies and percentages. Continuous variables were expressed as means and standard deviations. Paired *t*-tests were utilized to compare preoperative and postoperative anthropometric symmetry ratios (i.e., lip length, commissure length, nostril width, nostril breath). Statistical significance was established at a *p*-value of less than 0.05. All statistical analyses were performed using Stata software, version 17.0 (Stata Corp, College Station, TX, USA).

## 3. Results

### 3.1. Patient Characteristics

The present study included 188 consecutive patients who underwent ECLR at this institution, consisting of 104 (55.3%) males and 84 (44.7%) females. The average gestational age at birth was 38.6 ± 1.3 weeks, with 8 patients born preterm (defined as a gestational age (GA) less than 37 weeks). GA-corrected age was an average of 1.0 ± 0.5 months at the time of cleft lip repair. Patients weighed on average 4.1 ± 0.7 kg. Racial group breakdown consisted of 51.6% White/Caucasian, 10.1% Asian, 1.6% Black/African American, and 36.7% Other/Unreported race. Among these racial groups, 23.9% identified as Hispanic. Most patients were ASA class 2 (143, 76.1%) at the time of cleft lip repair and 45 (23.9%) patients were ASA class 1. Overall follow-up time following cleft lip repair was 3.1 ± 2.2 years across all patients (Table 1).

### 3.2. Cleft Characteristics

**Figure 3:**
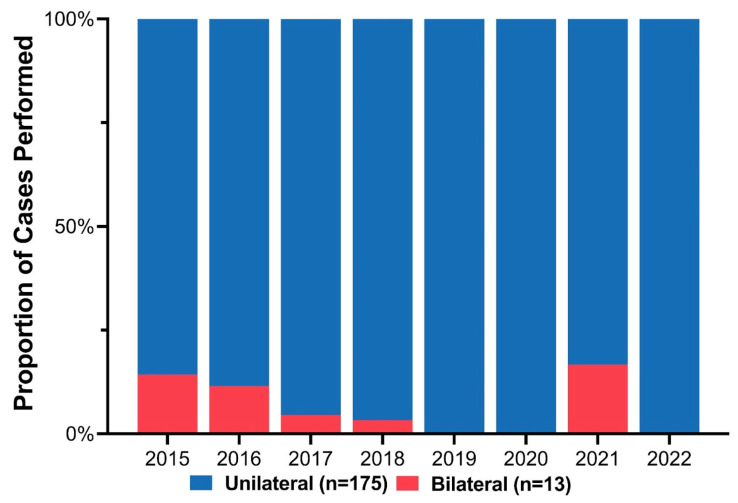
Annual proportion of unilateral versus bilateral cases among patients who underwent early cleft lip repair.

Cleft phenotypes included 96 cleft lip and palate (51.1%) and 92 isolated cleft lip (48.9%). Stratified based on cleft laterality, 13 (6.9%) patients had bilateral clefts, and the remaining 175 (93.1%) had unilateral clefts (109 (62.3%) left-sided, 66 (37.7%) right-sided). Initially, some patients with BCL/P deformities were repaired using the ECLR protocol. However, fewer BCL/P patients were included over the study period as the complication and revision rate exceeded the benefit of early repair (Figure 3). There were three patients with BCL/P who underwent ECLR more recently; however, all three cases had a complete cleft lip with a contralateral microform cleft lip and essentially underwent a procedure similar to unilateral cleft lip repair. Among the 175 unilateral clefts, the distribution of cleft severity included 84 (48.0%) incomplete, 82 (46.9%) complete, and 9 (5.1%) microform cleft lips (Table 1).

### 3.3. Intraoperative and Postoperative Complications

Four (1.7%) patients had postoperative complications following early cleft lip repair, which included two cases of nasal stent dislodgement, one case of wound dehiscence for a patient with BCL/P requiring revision, and one case of postoperative respiratory distress due to aspiration which resolved with no further complications. There were no anesthetic complications or perioperative mortality. For unilateral cases, the mean operative and anesthetic times were 120 ± 33 min and 189 ± 35 min, respectively. Bilateral clefts on average had a significantly longer operative time (170 ± 65 vs. 120 ± 33 min; *p* < 0.001) and anesthesia time (219 ± 42 vs. 189 ± 35 min; *p* < 0.005) compared to unilateral cases. Of note, patients with BCL/P with a microform cleft on one side had operative and anesthesia times comparable to unilateral clefts (anesthesia time: 203 ± 46 min; operative time: 115 ± 17 min). However, patients with BCL/P consisting of either complete clefts, incomplete clefts, or a combination of the two had the longest operations (anesthesia time: 224 ± 43 min; operative time: 189 ± 35 min). The mean hospital length of stay was 1.1 ± 1.4 days, which was comparable across all patients.

### 3.4. Secondary Lip Revision

**Figure 4:**
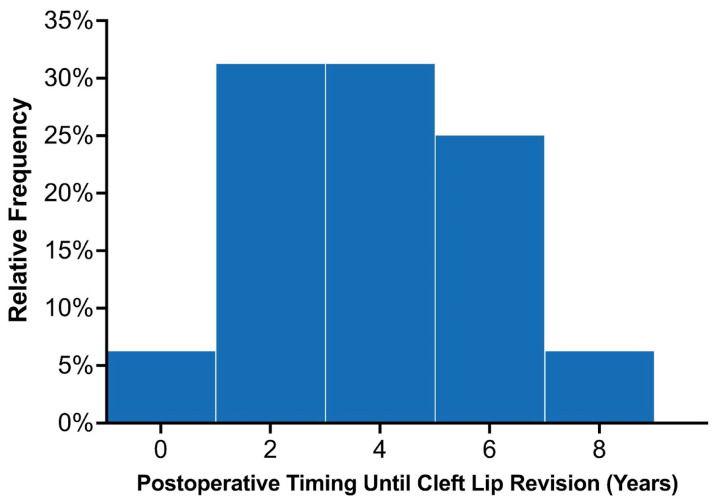
Distribution of the time elapsed from initial lip repair to secondary surgery among the twenty early cleft lip repair patients who required revisions.

Across the entire study population, 22 patients were recommended to undergo lip revision following their initial primary repair, of which 18 underwent surgical intervention. Among the 89 patients with at least 3 years of follow-up, 16 (18.0%) underwent lip revision following their primary repair. Common indications for minor revisions included vermillion asymmetry (n = 7), white roll misalignment (n = 5), and wet–dry junction asymmetry (n = 5; Table 2). The average time until lip revision postoperatively was 3.7 ± 1.9 years, (median: 4.1 years; interquartile range 2.0–5.1 years). The distribution of time to secondary surgery is demonstrated in Figure 4. Among the 80 patients with UCL/P with adequate follow-up, 14 (17.5%) underwent lip revision, most of which involved minor revisions (85.7%; n = 12/14). Among the 13 patients with BCL/P, two (15.4%) patients required complete takedown of the cleft lip, which were categorized as major revisions. These patients with BCL/P had a complete and incomplete cleft lip and underwent their primary repair in 2015. Among the three patients with BCL/P consisting of a microform cleft who underwent ECLR in 2021, one patient required minor revisions due to mild vermillion asymmetry. This patient was planned for a unilateral repair but was found to have a contralateral microform cleft intraoperatively, after which a staged cleft lip repair was planned for nine months later. A minor revision simultaneously given the patient was already undergoing surgical repair.

### 3.5. Anthropometric Outcomes

Among the 59 patients included in the photometric analysis, the average preoperative difference from ideal symmetry was 0.41 ± 0.16 for lip length and 0.63 ± 0.59 for commissure length, 1.17 ± 0.97 for nostril width, and 0.86 ± 0.79 for nostril breadth. Postoperatively, all anthropometric measurements demonstrated a significant improvement when compared to their respective preoperative values. This included lip length (0.13 ± 0.11; *p* < 0.001), commissure length (0.14 ± 0.12; *p* < 0.001), nostril width (0.23 ± 0.27; *p* < 0.001), and nostril breadth (0.16 ± 0.22; *p* < 0.001). All preoperative and postoperative anthropometric measurements are displayed in Table 3.

## 4. Discussion

**Figure 5:**
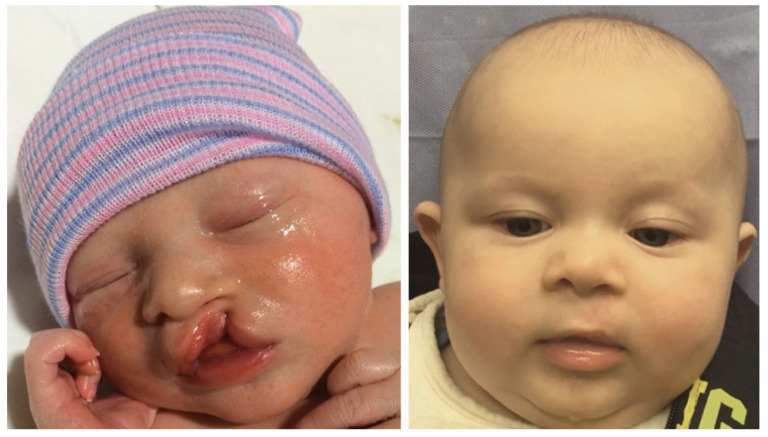
Preoperative (**left**) and three-month postoperative (**right**) photographs of a patient with left unilateral incomplete isolated cleft lip who underwent repair at two weeks of age. Reproduced with permission from Hammoudeh et al. [4], *Plastic and Reconstructive Surgery–Global Open*; published by Wolters Kluwer Health, Inc., 2017.

**Figure 6:**
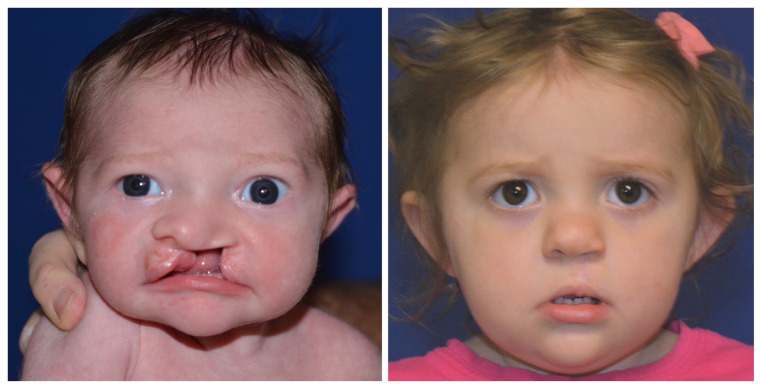
Preoperative (**left**) and 19 month postoperative (**right**) photographs of a patient with left unilateral complete cleft lip and palate who underwent repair at 18 days of age. Reproduced with permission from Wlodarczyk JR et al. [3], *Plastic and Reconstructive Surgery*; published by Wolters Kluwer Health, Inc., 2022.

**Figure 7:**
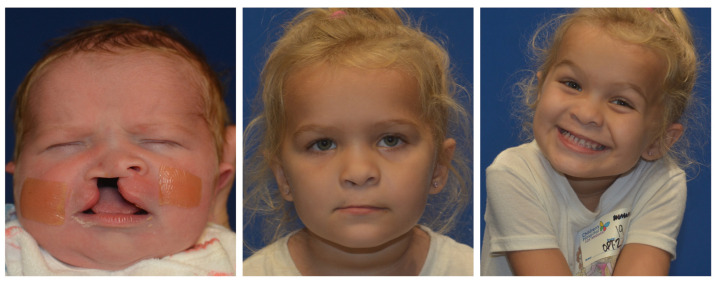
Preoperative (**left**), 42 month postoperative (**middle** and **right**) photographs of a patient with right unilateral complete cleft lip and palate who underwent repair at 13 days of age.

Reviewing the outcomes of 188 patients who underwent early cleft lip repair (ECLR), anthropometric findings demonstrate the ability of ECLR to enhance postoperative cleft lip symmetry and achieve malleability and molding of the nasal cartilage. In addition, the rates of surgical and anesthetic complications for ECLR were within traditional best practices [3,38]. This institution has been able to achieve excellent aesthetic outcomes with a revision rate that is substantially lower than those reported in traditional cleft lip repair in patients of varying cleft lip severity. Figure 5 shows preoperative and postoperative photographs of a patient with left unilateral incomplete isolated cleft lip who underwent repair at 14 days of life. Figure 6 and Figure 7 show similar photographs of two patients with unilateral complete cleft lip and palate who underwent repair at 18 days and 13 days of life, respectively. Beyond these improved anthropometric measurements, ECLR has been shown to optimize surgical scar healing, improve maternal–infant socializing, accelerate infant weight gain, promote normal feeding, and restore form and function as early as possible [2].

Furthermore, ECLR is beneficial since it eliminates the need for nasoalveolar molding (NAM) in these patients. NAM treatment protocols vary by institution; however, the commonality between these protocols is the gradual correction of the premaxilla, prolabial segment, and nostril with compliant appliance use. Significant heterogeneity exists in the specific protocols for adjustment and duration of NAM therapy (1 to 5 months) and follow-up protocols for NAM adjustments [39,40]. As mentioned previously, NAM can impose a significant burden of care on patients, their families, and the healthcare system. Its shortcomings include high cost, the necessity for biweekly in-office device adjustments, and stringent adherence to intricate at-home protocols. ECLR, in contrast, does not require these postoperative protocols and can alleviate some of the strain on families. Wlodarczyk et al. demonstrated that shifting the first 100 ECLR patients away from NAM led to an annual cost reduction of $2738 for families and $368,700 for the healthcare system [29]. Consequently, ECLR has the potential to improve access and adherence to care for financially and socially vulnerable populations. The reduced financial and time commitments with ECLR, compared to traditional lip repair with NAM, could negatively impact the quality of life of patients and parents. This, in turn, could have a negative effect on the child’s emotional development [41]. Moreover, the rates of breastfeeding in ECLR patients are comparable to the general population [42]. Breastfeeding can lead to increased maternal attachment, a particularly important aspect of caring for a child with significant health issues such as cleft deformities [43].

Another advantage of ECLR is improved patient outcomes, notably enhanced fetal weight gain [2]. A study contrasting ECLR with traditional cleft lip repair with NAM revealed a significant increase in weight gain among ECLR patients. This implies that patients undergoing traditional lip repair might endure unnecessarily prolonged feeding difficulties. These findings combined with other studies corroborate ECLR’s potential to attain levels of postoperative symmetry comparable to those that are achieved with NAM [3,4,10,11,18]. Collectively, these insights highlight the clear benefits of unilateral ECLR as an alternative to NAM, and other institutions should be encouraged to offer this protocol to families of cleft lip patients.

This cohort demonstrated a revision rate of 11.4% in unilateral CL patients, most of which were minor revisions. Revision rates following traditional lip repair have been reported to be approximately 28%, although may range from 0% to 57% or higher [44,45,46]. These results demonstrate a reduced need for secondary revisional procedures compared to the reported rates in some of the existing literature and this institution’s previous historical rates. Additionally, previous studies have noted comparable revision rates of traditional primary unilateral cleft lip repair between 3 and 6 months and ECLR before five weeks of age [11,47]. For patients with UCL/P, future studies should further investigate revision rates in patients with UCL/P undergoing traditional versus early cleft lip repair to further elucidate differences between traditional cleft lip repair and ECLR protocols and better support these initial findings presented by this institution [11].

Importantly, bilateral cleft lip repair patients were initially included in the ECLR protocol. However, after considering the higher rates of complications and revisions that already exist for bilateral cleft lip patients [48], traditional cleft lip repair protocols involving presurgical NAM were deemed the most appropriate for this patient population. Bilateral cleft lip patients have unique features such as protruding premaxilla, which often require NAM for aligning soft tissue and bony segments. Additionally, bilateral cleft lips, in particular, pose a reconstructive challenge given the lack of orbicularis muscle in the prolabial segment. Given these findings, NAM therapy and the standard cleft lip repair timeline is recommended for patients with complete/incomplete bilateral cleft lip deformities, a protuberant premaxilla, and a short prolabial segment.

Prior to implementing an ECLR protocol at any institution, a care team must diligently build upon the infrastructure needed to provide the required services to patients and their families. Careful patient selection is imperative, as it forms the backbone of safe surgical proceedings. This institutional protocol offers ECLR to patients with an ASA class below III. If the surgery is deemed potentially hazardous due to the patient’s underlying health conditions, it is prudent to postpone the surgical intervention until the patient reaches a state of medical stability. Proper timing of cleft lip repair also requires routinely adjusting for gestational age in patients born prematurely. Based on the stability of the premature infant, it is advisable to postpone surgical repair until gestational age is in line with the ECLR timeline.

This institution’s surgical recommendations have also been refined based on experience. Surgically, the initial incision is made, local anesthesia is administered, and the aberrant attachments of the orbicularis oris are liberated. The local anesthesia with vasoconstrictor allows for better visualization of the orbicularis muscle prior to incising the muscle from its original, abnormal attachment. Another key lesson is that the use of a tapered needle, as opposed to a cutting or reverse-cutting needle, minimized tissue trauma.

It is important to acknowledge nasal stent dislodgement as a possible postoperative complication, as was experienced by two patients in this cohort. Best practice commences intraoperatively by ensuring the nasal stent is firmly secured to prevent premature dislodgement. Furthermore, it is imperative to comprehensively educate the patient’s family on maintaining nasal stent cleanliness and patency. Caretakers should be encouraged to voice any concerns regarding the potential loosening of the stent, thereby promoting proactive and informed care.

Regarding anesthetic use, the potential implications of infant exposure to general anesthesia prompted two national landmark studies. The initial approach to anesthesia was based on a specialized protocol that eliminated the use of inhaled sevoflurane. However, both the GAS and PANDA studies found no long-term neurocognitive sequelae from general anesthesia exposure before three months of age prompting a transition to general anesthesia use in this ECLR protocol [34,35,36,49].

### Limitations and Future Directions

While this study demonstrates promising insights that seek to improve clinical outcomes, potential limitations do exist. These include the relatively small sample size at a single institution and lack of randomization. Additionally, patients who have undergone early repair have yet to be compared to patients who undergo traditional lip repair at three to six months of age. A comprehensive comparison of the two groups assessing for postoperative outcomes and anthropometric symmetry is warranted. Long-term assessment of the early cleft lip repair protocol will be determined with the long-term neurocognitive outcomes of these patients, anthropomorphic changes in the lip and nose as the patients age, and the need for future secondary procedures. Continued evaluation of the ECLR protocol’s efficacy and the neurocognitive effects of early anesthesia as patients mature is encouraged. Thus, the future direction of this study will aim to explore differences in revision rates, the impact of ECLR on the timing of palatal repair, indication for cleft lip rhinoplasty at skeletal maturity, and long-term aesthetic outcomes of ECLR versus traditional lip repair.

## 5. Conclusions

This study provides an updated review of this institution’s eight years of experience with early cleft lip repair. The paradigm shift to earlier cleft lip management has been highly effective with improved postoperative nasal and lip symmetry, optimized scar healing, and a safety profile within the standard of care. For patients with bilateral cleft lip and nasal deformities, traditional lip repair should be performed between three and six months of age, with the utilization of NAM depending on the clefts’ severity. While further research is encouraged, early cleft lip repair continues to gain acceptance as an option for families that want to avoid nasoalveolar molding and desire early repair.

## Figures and Tables

**Table 1:** Demographics, medical history, and cleft characteristics of patients who underwent early cleft lip repair.

Patient Characteristic			Total n (%)
Total Number of Patients			188 (100.0%)
*Sex*			
	Male		104 (55.3%)
	Female		84 (44.7%)
*Race*			
	White/Caucasian		97 (51.6%)
	Asian		19 (10.1%)
	Black/African American		3 (1.6%)
	Other/Unreported		69 (36.7%)
*Ethnicity*			
	Hispanic		52 (27.7%)
Average GA-Corrected Age at Surgery			1.0 ± 0.5 Month
Average Weight at Surgery			4.1 ± 0.7 kg
*ASA Classification*			
	I		45 (23.9%)
	II		143 (76.1%)
Pre-Term Birth			8 (4.3%)
*Cleft Phenotype*			
		Isolated Cleft Lip	89 (50.3%)
		Cleft Lip and Palate	88 (49.7%)
*Cleft Laterality*			
	Unilateral		173 (93.1%)
		Left	109 (62.3%)
		Right	66 (37.7%)
		Bilateral	13 (6.9%)
*Cleft Severity*			
		Complete	84 (48.0%)
		Incomplete	82 (46.9%)
		Microform	9 (5.1%)
Follow-Up Time			3.1 ± 2.2 Years

Abbreviations: ASA: American Society of Anesthesiologists; GA: Gestational Age.

**Table 2:** Indications for cleft lip revision surgery among patients who underwent early cleft lip repair.

Patient Characteristic			Total n (%)	Unilateral n (%)	Bilateral n (%)
Total Number of Patients			n = 188	n = 175	n = 13
Total Lip Revisions			22 (11.7%)	20 (11.4%)	2 (15.4%)
	*Major Revisions*		6 (3.2%)	4 (2.3%)	2 (15.4%)
		Complete Takedown	5 (2.7%)	3 (1.7%)	2 (15.4%)
		Wound Dehiscence	1 (0.5%)	0 (0.0%)	1 (7.7%)
	*Minor Revisions*		16 (8.5%)	16 (9.1%)	0 (0.0%)
		Vermillion Asymmetry	10 (5.3%)	10 (5.7%)	0 (0.0%)
		White Roll Misalignment	8 (4.3%)	8 (4.6%)	0 (0.0%)
		Wet–Dry Junction Asymmetry	8 (4.3%)	8 (4.6%)	0 (0.0%)
		Short Lip	4 (2.1%)	4 (2.3%)	0 (0.0%)
		Intraoral Excess	3 (1.6%)	3 (1.7%)	0 (0.0%)
		Hypertrophic Scar	1 (0.5%)	1 (0.6%)	0 (0.0%)

Patients could have had more than one indication for a single secondary revision surgery.

**Table 3:** Anthropometric comparison of preoperative versus postoperative distance from ideal symmetry for patients who underwent early cleft lip repair.

Anthropometric Distance from Ideal Symmetry	Preoperative	Postoperative	*p*-Value
Lip length ^1^	0.41 ± 0.16	0.13 ± 0.11	<0.001 *
Commissure length ^2^	0.63 ± 0.59	0.14 ± 0.12	<0.001 *
Nostril width ^3^	1.17 ± 0.97	0.23 ± 0.27	<0.001 *
Nostril breadth ^4^	0.86 ± 0.79	0.16 ± 0.22	<0.001 *

Symmetry ratios were calculated by taking the absolute anthropometric measurement of the cleft side and dividing it by the contralateral non-cleft side. This value was then subtracted by 1.00, which was defined as ideal symmetry ratio. ^1^ Lip length was defined as the distance from the lateral aspect of the columellar base to the ipsilateral peak of Cupid’s bow. ^2^ Commissure length was defined as the distance from the labial commissure to the trough of Cupid’s bow. ^3^ Nostril width was defined as the distance from the columellar base at its midpoint to the inner nostril at its most inferolateral point). ^4^ Nostril breadth was defined as the distance from the lateral aspect of the columellar base to the widest point of the nasal alae. * indicates statistical significance at *p*-value less than 0.05.

## Data Availability

The data presented in this study are available upon request from the corresponding author. The data are not publicly available since certain data are subject to further research.
